# Supplementary figures and images for: Reducing lipid bilayer stress by monounsaturated fatty acids protects renal proximal tubules in diabetes
Source: eLife. 2022 May 12;11:e74391. doi: 10.7554/eLife.74391 (PMC9154741; doi:10.7554/eLife.74391)

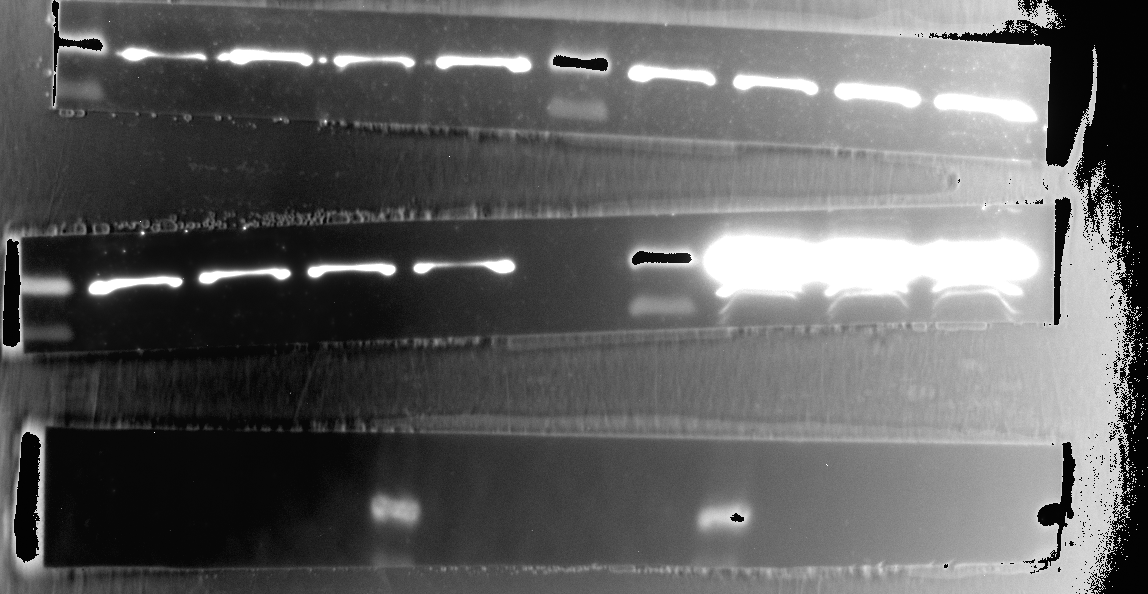

Supplement: Figure 3—figure supplement 1—source data 1. [file elife-74391-fig3-figsupp1-data1.zip › Figure 3ΓÇôfigure supplement 1ΓÇôsource data 1/GAPDH+Marker_raw.tif]

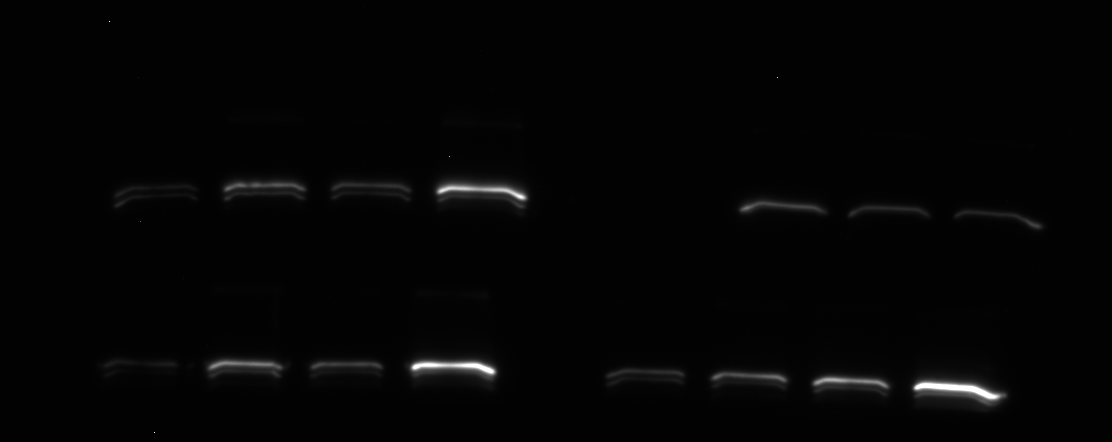

Supplement: Figure 3—figure supplement 1—source data 1. [file elife-74391-fig3-figsupp1-data1.zip › Figure 3ΓÇôfigure supplement 1ΓÇôsource data 1/p62_raw.tif]

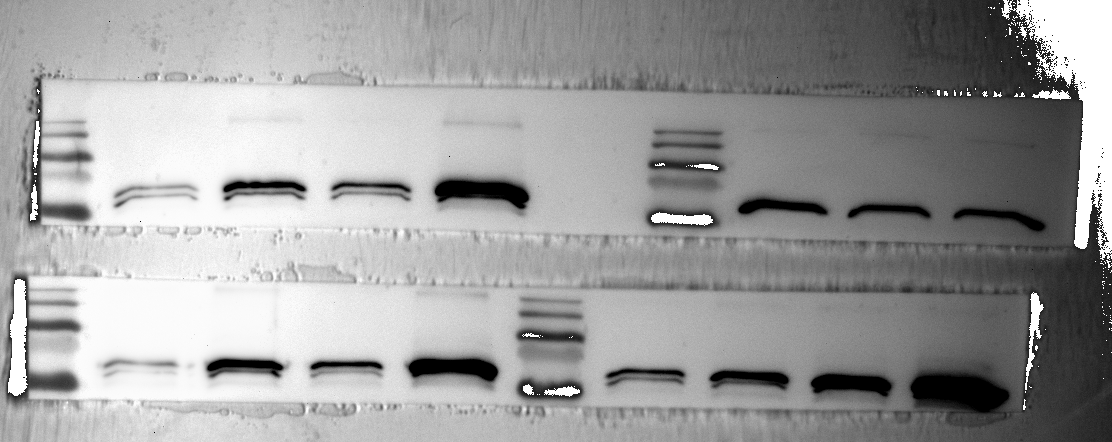

Supplement: Figure 3—figure supplement 1—source data 1. [file elife-74391-fig3-figsupp1-data1.zip › Figure 3ΓÇôfigure supplement 1ΓÇôsource data 1/P62+Marker_raw.tif]

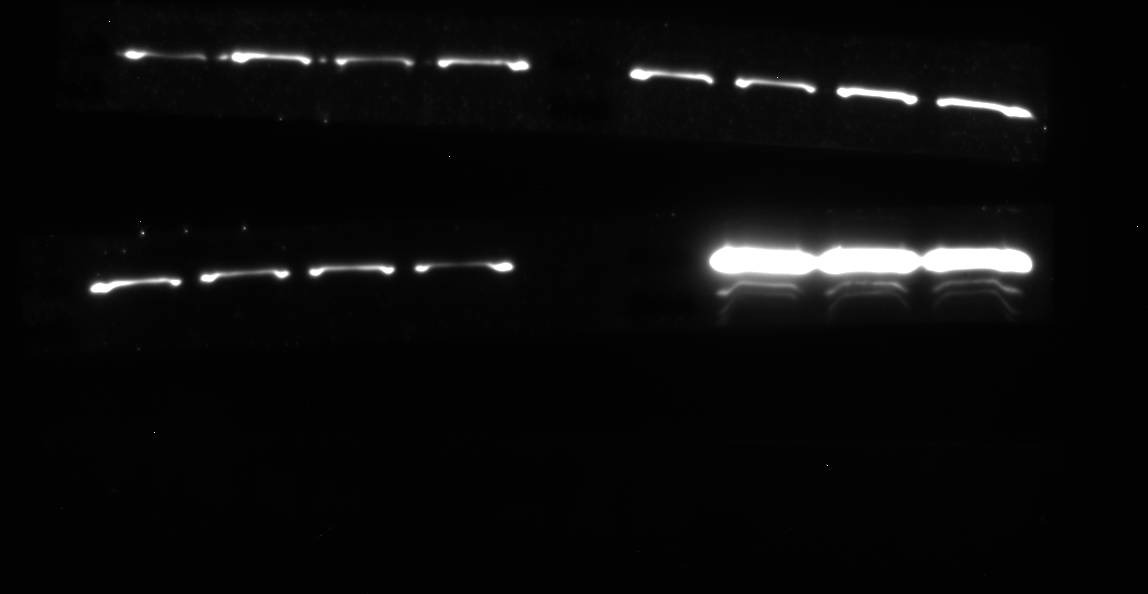

Supplement: Figure 3—figure supplement 1—source data 1. [file elife-74391-fig3-figsupp1-data1.zip › Figure 3ΓÇôfigure supplement 1ΓÇôsource data 1/GAPDH_raw.tif]

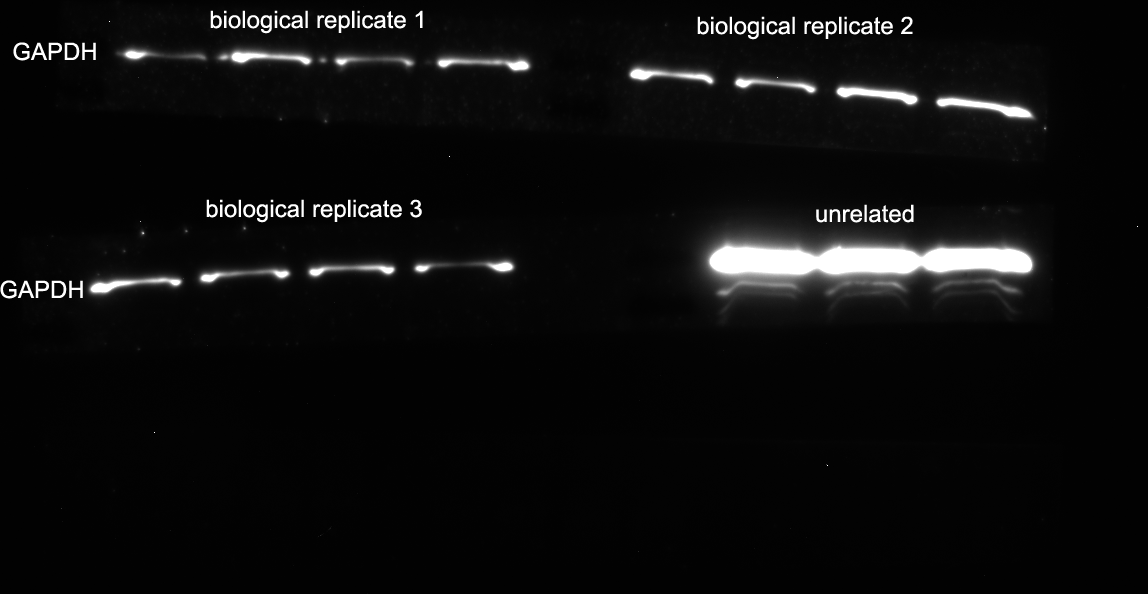

Supplement: Figure 3—figure supplement 1—source data 1. [file elife-74391-fig3-figsupp1-data1.zip › Figure 3ΓÇôfigure supplement 1ΓÇôsource data 1/GAPDH_Labels.tif]

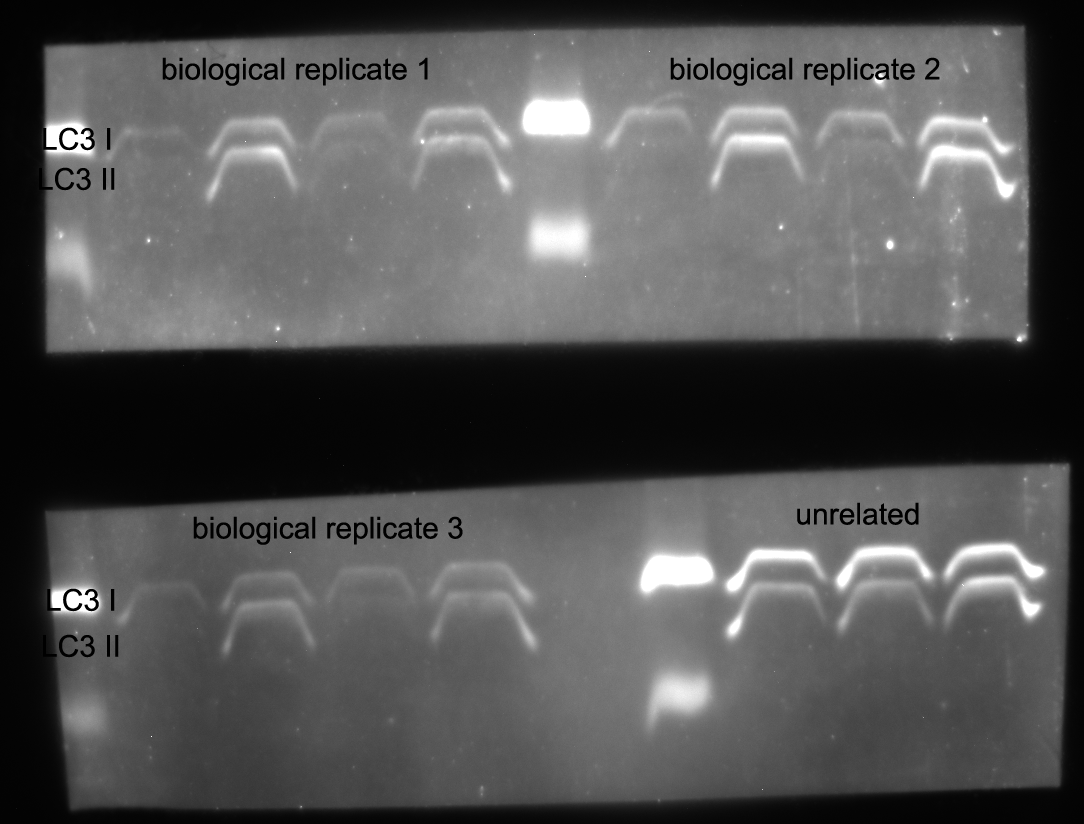

Supplement: Figure 3—figure supplement 1—source data 1. [file elife-74391-fig3-figsupp1-data1.zip › Figure 3ΓÇôfigure supplement 1ΓÇôsource data 1/LC3 low exposure_labels.tif]

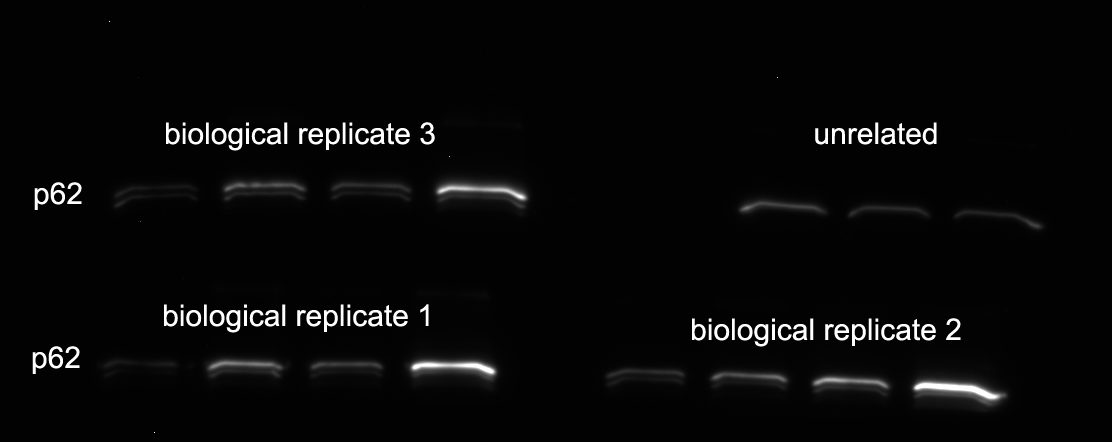

Supplement: Figure 3—figure supplement 1—source data 1. [file elife-74391-fig3-figsupp1-data1.zip › Figure 3ΓÇôfigure supplement 1ΓÇôsource data 1/p62_Labels.tif]

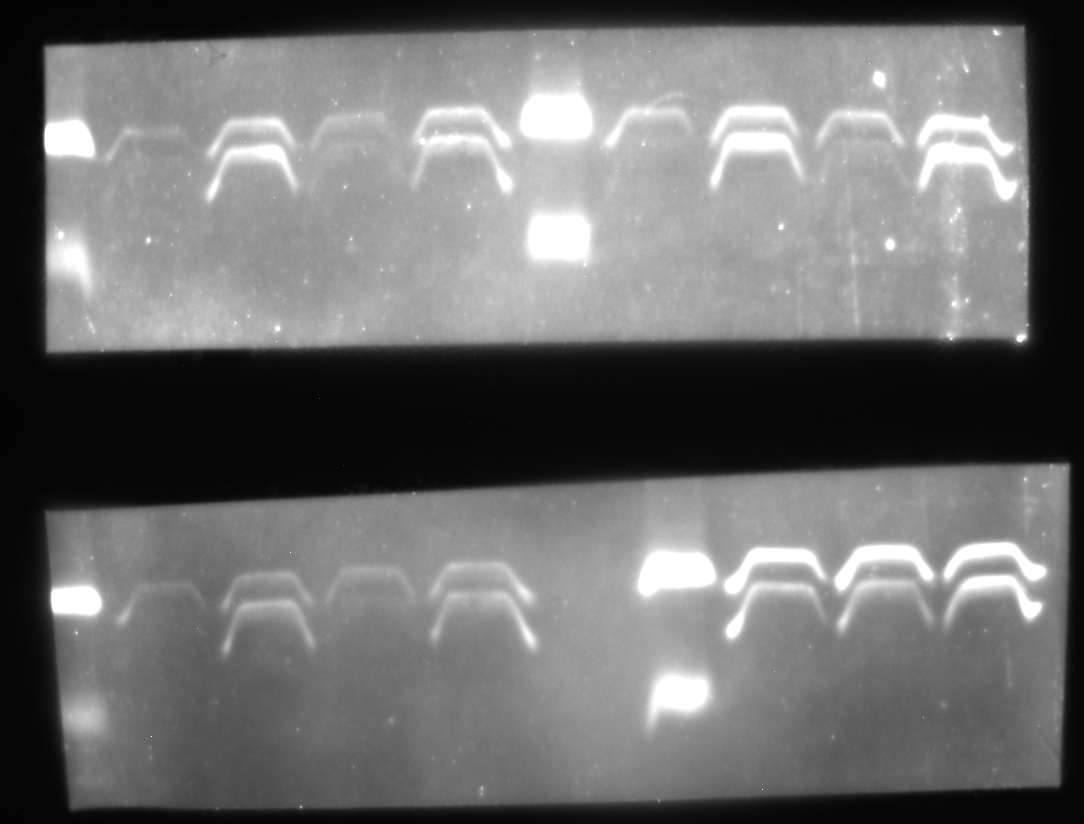

Supplement: Figure 3—figure supplement 1—source data 1. [file elife-74391-fig3-figsupp1-data1.zip › Figure 3ΓÇôfigure supplement 1ΓÇôsource data 1/LC3 long exposure_raw.tif]

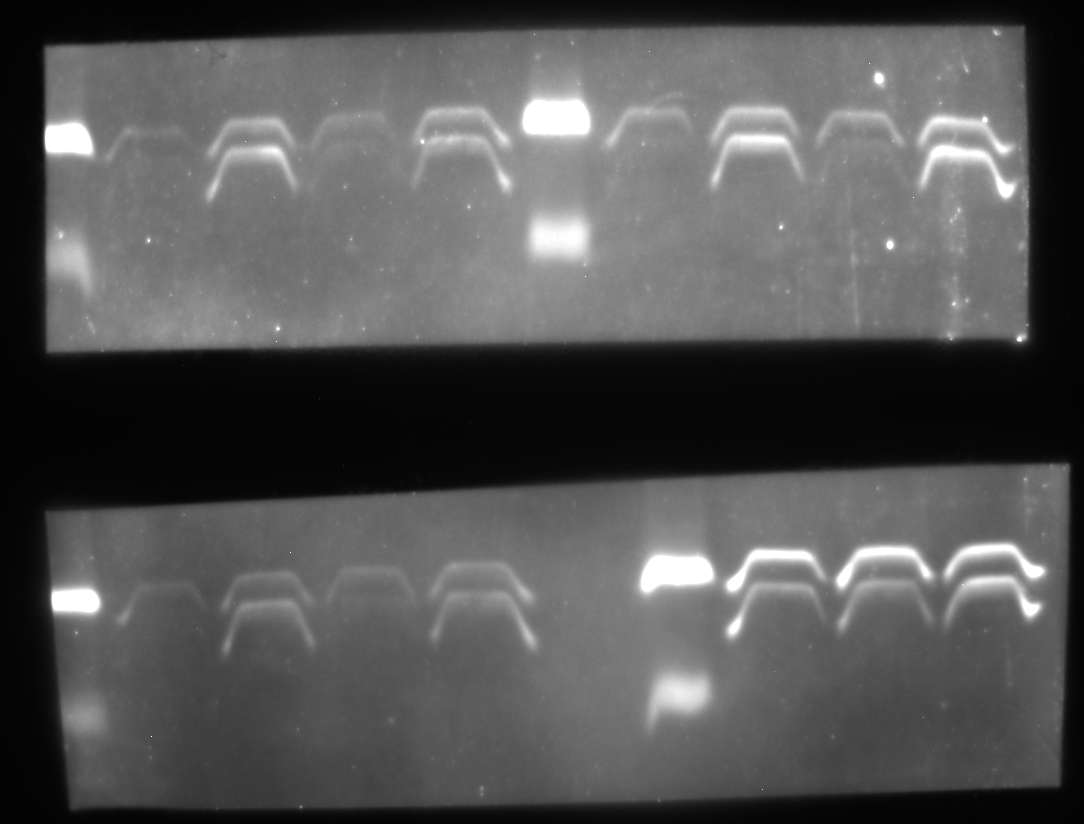

Supplement: Figure 3—figure supplement 1—source data 1. [file elife-74391-fig3-figsupp1-data1.zip › Figure 3ΓÇôfigure supplement 1ΓÇôsource data 1/LC3 low exposure_raw.tif]
